# Professional competence development in the context of clinical uncertainty: a qualitative exploration of infection prevention and control nurses at a tertiary hospital in China

**DOI:** 10.3389/fpubh.2026.1865842

**Published:** 2026-09-07

**Authors:** Qiudi Song, Qianqian Li, Li Zhang, Min Bao, Kun Zhou, Qiao Lu, Xixi Liang, Jing Mu, Fang Liu

**Affiliations:** 1 Department of Intensive Care Medicine, Nanjing Drum Tower Hospital Group Suqian Hospital, Suqian, China; 2 Department of Gynecology, Nanjing Drum Tower Hospital Group Suqian Hospital, Suqian, China; 3 Department of Public Health, Nanjing Drum Tower Hospital Group Suqian Hospital, Suqian, China; 4 Department of Hepatobiliary Surgery, Nanjing Drum Tower Hospital Group Suqian Hospital, Suqian, China; 5 Interventional Oncology and Rehabilitation Ward, Nanjing Drum Tower Hospital Group Suqian Hospital, Suqian, China; 6 Department of Orthopedic Trauma, Nanjing Drum Tower Hospital Group Suqian Hospital, Suqian, China; 7 Department of Spine Surgery, Nanjing Drum Tower Hospital Group Suqian Hospital, Suqian, China; 8 School of Education, Macquarie University, Sydney, NSW, Australia

**Keywords:** hospital infection, infection prevention and control nurses, nursing practice, professional competence, qualitative research

## Abstract

**Background:**

Infection prevention and control nurses (IPCNs) are critical to preventing and controlling of healthcare-associated infections (HAIs), supporting patient safety, and responding to public health disasters. Although the significance of such a role is increasingly recognized, China lacks a unified training program or credentialing for IPCNs. We are now in a highly contrived and uncertain clinical setting that urges us to acquire a deep-seated comprehension of how we may enable IPCNs to develop their professional competence without institutionalized support. It is also necessary to identify the contextual and structural challenges they encounter.

**Objectives:**

To explore the professional dilemmas facing IPCNs in a Chinese tertiary hospital setting, characterized by a lack of standardized training systems and institutional support, and how they navigate these dilemmas in dynamic and complex clinical scenarios. The study also aimed to examine how these experiences shape the development of professional competence and its relationship with professional role identity.

**Methods:**

This study was conducted between 01/10/2025 and 30/11/2025 at a Grade A tertiary hospital in Jiangsu Province, China, where IPCNs with diverse professional backgrounds and levels of experience were recruited. Data were collected through online semi-structured interviews, with 16 participants. The interview guide focused on the professional dilemmas IPCNs experience in clinical practice, how they address these dilemmas, and how they develop their professional competency. All interviews were audio-recorded with the participants’ consent, and the recordings were transcribed verbatim. A thematic analysis of the transcripts was performed to identify and synthesize recurring and meaningful themes.

**Results:**

Based on the findings, four core themes and 14 subthemes were identified: (1) practice environments characterized by prominent uncertainty; (2) challenges and costs in achieving competent practice; (3) informal learning in the absence of institutional support; (4) mutual shaping of professional competence and role identity.

**Conclusion:**

This study provided empirical evidence of the dynamic process of professional competency development among IPCNs at a tertiary hospital in China, and clarified how this process was interwoven with the construction of their professional role identity in uncertain clinical contexts lacking systematic training and institutional support. These findings offer transferable insights for developing contextually appropriate IPCN training systems, support mechanisms, and competency development pathways in similarly structured tertiary-hospital settings.

## 1 Introduction

Infection prevention and control nurses (IPCNs) are specialist nursing professionals appointed by healthcare institutions to monitor and control healthcare-associated infections (HAIs) (1). IPCNs play a vital role in healthcare institutions. Their work has proven invaluable in reducing HAIs and responding to public health emergencies, such as the current pandemic caused by the SARS-CoV-2 virus. IPCNs can reduce the incidence of HAIs by approximately 50% by implementing and supervising infection prevention and control measures. They can also contribute to delivering cost-effective healthcare (2, 3). Further studies have indicated that effective interventions by IPCNs significantly reduce healthcare-associated infection rates, improve patient safety, and promote adherence to standardized infection prevention and control procedures in healthcare facilities (4, 5). In infection prevention and control (IPC) practices, the effective fulfillment of IPCNs’ professional roles depends heavily on solid and comprehensive professional competencies (6). IPCNs’ competencies do not occur spontaneously. Instead, it needs institutional support and systematic training at an organizational level. There were also implications of the long-term strategic plan of the Association for Professionals in Infection Control and Epidemiology (APIC) in 2012 on the role of professional competency development and certification systems for IPCNs at an organizational level, acting as the development (7). Fukuda et al. (8) postulated a strong correlation between patient safety and infection control activities in the hospital. Thus, healthcare institutions must ensure patient safety by developing IPCNs’ professional competencies and providing institutional reinforcement.

Professional competency training for IPCNs and institutional support to foster their growth have become a vital concern in recent years, in light of international policy documents and professional guidelines, to secure the quality of healthcare-associated infection prevention and control, as well as patient safety. In addition, some parts of the world, such as Europe and North America (9–11), have established clear prerequisites that require IPCNs to complete standardized training and a defined period of clinical practice before certification. In contrast, the development of IPCNs in China began later and has a shorter history. Although relevant policies and professional guidelines have been continuously refined, China has not established a nationally standardized institutional training framework and qualification certification system (12). To date, IPCN professional competency development in China has largely relied on individual experience and internal arrangements within healthcare institutions. In China’s complex and dynamic clinical environment, significant variations exist in the management and utilization of IPC resources, organizational support, workload, staffing levels, and role positioning across regions and healthcare institutions. This contributes to more pronounced heterogeneity in the professional competency development of IPCNs (13, 14). Research on IPCN competency by both domestic and international scholars nowadays primarily focuses on the following aspects. Firstly, to develop competency frameworks, studies have created core competency frameworks by defining the essential elements of competency required for IPCNs to fulfill their professional roles. This standardizes training content and supports the assessment of IPCNs’ professional competency (15–17). Secondly, to develop competency assessment tools for IPCNs, studies have produced evaluation instruments for education and training based on theoretical models, in order to determine whether IPCNs’ professional competency levels are adequate for fulfilling their role responsibilities (18, 19). Finally, regarding IPCN competency development, studies have used interventions such as continuing education (20) and simulation (21) to help develop professional competency. This supports IPCNs in clinical practice and improves their ability to fulfill their roles. These studies have provided an important foundation for understanding IPCNs’ professional competency. However, they have largely been conducted in stable practice contexts, assuming clearly defined role boundaries. There has been insufficient attention paid to how the professional competency of IPCNs is formed and developed in clinical contexts characterized by a lack of institutional support and uncertainty. Role identity also plays a significant role in the development of professional competence. Professional competence is formed not only through knowledge and skills training, but also through role identity (22, 23). Previous research has identified a close relationship between role identity and professional competence (24). However, discussions regarding the relationship between the two remain relatively scarce in studies related to IPCNs.

Therefore, to develop a comprehensive understanding of how professional competence evolves among IPCNs in a Chinese tertiary hospital setting, in the absence of standardized training systems and clearly defined role boundaries, it is crucial to draw on the first-hand experiences of frontline clinical IPCNs. This will provide in-depth insights into their personal experiences, challenges and adaptive strategies within real-world practice settings. Accordingly, this study used semi-structured interviews to explore how IPCNs develop professional competence in uncertain clinical practice contexts, the structural conditions that shape this process and the relationship between professional competence and role identity. The aim was to provide a theoretical basis for developing more contextually adaptive training systems, continuous support mechanisms and policies.

## 2 Methods

### 2.1 Design

This study adopted a qualitative descriptive research design through online semi-structured interviews with IPCNs engaged in IPC at a Grade A tertiary general hospital in Jiangsu Province, China. This study examined the professional dilemmas and coping strategies healthcare professionals encountered in practice contexts characterized by a lack of unified, institutionalized training and ambiguous role boundaries. It also explored how these experiences supported the development of their professional competence. In addition, the design, conduct, and reporting of this study strictly adhered to the Consolidated Criteria for Reporting Qualitative Research (COREQ) (25), and the completed checklist is provided in Supplementary File 1.

### 2.2 Participants

In this study, purposive sampling was used to recruit participants at a Grade A tertiary general hospital located in Suqian, Jiangsu Province, China, between October and November 2025. To maximize sample diversity, this research sampled individuals with different work experience levels, educational attainment, and professional ranks. Inclusion criteria included: (1) registered and current IPCNs; (2) a minimum of 1 year of experience as an IPCN, consistent with thresholds used in previous IPC research, as nurses with less experience are still adjusting to the role (26, 27), and (3) volunteering to be included in this study. The exclusion criteria were: (1) IPCNs on leave, on vacation, or on rotational assignment; and (2) those who could not attend the entire interview process because of personal reasons. The researcher sent invitation letters to potential participants via email. These letters were accompanied by detailed online clarifications of the study objective, the interview content, participation requirements, measures to guarantee confidentiality, possible risks, and the right to withdraw. Participants also signed written informed consent forms after being fully informed. In total, 16 participants joined this study.

### 2.3 Data collection

Qualitative data were collected through one-to-one semi-structured in-depth interviews in this study. Semi-structured interviews combine systematicity and flexibility, allowing participants to elaborate freely based on their experiences while ensuring coverage of core research questions, yielding data with greater depth and contextual richness. The interview guide was developed based on the research objectives and preliminary findings (28). The guide’s content was refined through repeated discussions and revisions. Two qualitative research experts, each with over 5 years’ experience in IPC, were also invited to evaluate its content validity. The research team also conducted pilot interviews with two IPCNs to test the questions’ logical coherence and comprehensibility. The feedback obtained was used to optimize the interview process but was not included in the formal data analysis. The final interview guide consisted of the following questions: (1) Could you describe how you began to become competent in your role as an IPCN? (2) What difficulties have you encountered in your daily practice, and how did you address them? (3) Through what experiences did you gradually acquire the competencies required for an IPCN? (4) In your view, what forms of support or learning approaches were most helpful to you during the process of competency development? (5) How do you think your perception of your role as an IPCN has changed as your competencies have improved?

To ensure the privacy and professionalism of the interviews, all interviews were conducted via an online encrypted platform. The first author (QS), a female registered IPCN with a bachelor’s degree in nursing and formal training in qualitative research, personally conducted all interviews. No prior relationship was established with the participants before recruitment, and all interviews were audio-recorded throughout with participants’ written consent. All interviews were conducted in Mandarin Chinese, and transcribed verbatim by QS within 48 h of each interview. QLu cross-checked transcripts against the original audio recordings to ensure accuracy. Coding and theme development were conducted in Chinese to preserve the original meaning and nuance of participants’ accounts. Only the illustrative quotations selected for presentation in the manuscript were translated into English by a bilingual member of the research team (JM), who is fluent in both Chinese and English. A second bilingual researcher (FL) independently reviewed the translated quotations against the original Chinese transcripts to confirm that the intended meaning was preserved before inclusion in the manuscript. Each interview lasted between 30 and 60 min, with the length adjusted as necessary to allow participants to fully express themselves. The team conducted 16 formal interviews. Preliminary analysis confirmed that data saturation was reached during the 15th and 16th interviews, meaning that no new significant themes or perspectives emerged from the additional data. No participants withdrew during the entire data collection process. All participants signed an informed consent form and provided their contact details for any necessary subsequent follow-up or member checking. Meanwhile, this study gave full consideration to research ethics. All data were kept strictly confidential during collection and storage, and participants’ identities were protected through anonymization to ensure compliance and the trustworthiness of the research process.

### 2.4 Data analysis

This study adopted Braun and Clarke’s thematic analysis framework (29), applying a coding-consensus approach in which three researchers (JM, QS, and QL), independently coded the data before reconciling discrepancies through discussion. This approach was chosen to enhance coding consistency and auditability across a relatively structured set of interview questions. Coding was primarily inductive, with codes generated from participants’ accounts rather than from a predetermined theoretical framework or coding scheme (29). The thematic analysis followed six phases: (1) Familiarizing with the data: The research team members read all the interview transcripts so that they came to a comprehensive understanding of what was being said by the participants. (2) The production of first codes: We used a line-by-line open coding technique in order to find out the important words, cues of action and implicit meanings of the data. This enabled us to develop a provisional coding structure. (3) Themes search: Codes that were similar were grouped and united into primary themes, and the links among various codes were examined. (4) Themes review: Themes were evaluated and refined for their representativeness and logical arrangement in the various transcripts. (5) Naming and defining themes: Themes were named and defined, respectively, and had a definition that was more related to what the research entailed. (6) Generating the report: In reporting and interpreting the data, the initial snippets were strictly connected to the research questions in order to make sure that the themes were true to the experiences of the participants. The thematic framework was also discussed by the research team throughout the process and cross-checked to eventually agree on all core thematic and sub-themes. This increased the study’s validity and analytical value. All textual data were organized, coded, and managed using NVivo 14 software.

### 2.5 Rigor

To ensure the rigor and trustworthiness of this study, the research team applied the four evaluation criteria proposed by Guba and Lincoln (30), namely credibility, dependability, confirmability, and transferability, to conduct quality control and ensure the rigor throughout the entire research process. (a) Credibility: During the data analysis process, the research team implemented investigator triangulation, whereby multiple researchers with backgrounds in IPC management, as well as qualitative research, conducted coding and theme identification independently. When discrepancies arose, researchers returned to the original transcripts to re-examine the contextual meaning of the disputed segments and resolved them through discussion until they reached consensus. In addition, some participants were invited to take part in a process called ‘member checking’, which involved verifying whether the researchers’ interpretations were consistent with the participants’ original perspectives. This enhanced the accuracy of the data interpretation. (b) Dependability: The study’s reproducibility was enhanced by clearly documenting the research process, instrument use, and data processing procedures. In addition, a researcher specializing in IPC and qualitative research was invited to independently review and audit a portion of the interview data and the analytical process. This ensured the stability and logical rigor of the research process and helped ensure that comparable results can be obtained under similar conditions. (c) Confirmability: To minimize the influence of the researchers’ subjective bias on the research findings, a rigorous audit trail was maintained throughout the entire research process. This included interview transcripts, initial coding sheets and records of the categorization of themes. (d) Transferability: This study provided a detailed description of the research context, participant recruitment strategies, and data collection and analysis methods.

### 2.6 Ethical consideration

The Ethics Committee of Suqian Hospital, Nanjing Drum Tower Hospital Group approved the study proposal (2023-065-02). All methods were performed in accordance with the Declaration of Helsinki. Participants were guaranteed anonymity, confidentiality and the right to withdraw at any time. All audio recordings were saved on a password-protected computer, while all paper materials were stored in a locked cabinet under the supervision of designated personnel.

## 3 Results

A total of 16 IPCNs were recruited for this study, all of whom completed the full interviews. Table 1 provides a detailed description of the participants’ demographic characteristics and professional backgrounds using frequencies, percentages, and means (standard deviations).

**Table 1:** Participant characteristics (*N* = 16).

Variables	Total (*N* = 16)
Age (years)	
Mean ± SD	40.44 ± 7.35
Gender *N* (%)	
Male	2(12.5%)
Female	14(87.5%)
Education *N* (%)	
Bachelor’s degree	11(68.75%)
Master’s degree	5(31.25%)
Doctoral degree	-
Professional title *N* (%)	
Junior	4(25.0%)
Intermediate	6(37.5%)
Senior	6(37.5%)
Years of full-time experience as an IPCN (years)	
Mean ± SD	11.88 ± 6.88
Involvement in infection control management or training responsibilities *N* (%)	
Yes	11(68.75%)
No	5(31.25%)

In China’s nursing professional title system, titles are awarded based on a combination of academic qualifications, years of clinical practice, performance in title examinations, and professional competency assessments (49). Titles are ranked, from lowest to highest, as follows: Staff Nurse and Nurse Practitioner are classified as junior titles; Nurse-in-charge is classified as an intermediate title; and Associate Chief Nurse and Chief Nurse are classified as senior titles.

The research used thematic analysis, producing four overarching themes and 14 subthemes (Table 2) that clearly demonstrate the issues and coping mechanisms among Chinese IPCNs in the context of inadequate institutional training, vague role specifications, and unclear practice conditions. The study also highlights the facilitating role of these coping experiences in building their professional competence. The four core themes include: “Practice environments characterized by prominent uncertainty,” “Challenges and costs in achieving competent practice,” “Informal learning in the absence of institutional support,” and “Mutual shaping of professional competence and role identity.”

**Table 2:** Overview of the themes and sub-themes.

Theme	Sub-themes
Practice environments characterized by prominent uncertainty	Uncertainty in role positioning
	Uncertainty in work-related stress
	Uncertainty in risk exposure
Challenges and costs in achieving competent practice	Unclear initial competence boundaries
	Ambiguity in practical judgment
	Role insecurity
	Sustained personal depletion
Informal learning in the absence of institutional support	Peer mentoring
	Experiential learning
	Compensatory competence development
	Developing a sense of competence through practice
Mutual shaping of professional competence and role identity	Competence experience-driven role identity transformation
	Mutual reinforcement of competence accumulation and role identity
	Role identity destabilization upon competence setbacks

### 3.1 Practice environments characterized by prominent uncertainty

The professional competence of the IPCNs in this study developed progressively within complex healthcare contexts characterized by ambiguous role boundaries, sustained work-related stress, complex job demands and unpredictable clinical risks. This core theme encompasses three subthemes: “Uncertainty in role positioning,” “Uncertainty in work-related stress,” and “Uncertainty in risk exposure.”

#### 3.1.1 Uncertainty in role positioning

IPCNs frequently encounter issues such as ambiguous role positioning and unclear responsibility boundaries during clinical decision-making because of a lack of clear role positioning and well-defined, systematic evaluation criteria. This structural uncertainty impedes their ability to accurately evaluate how well their practice aligns with role expectations, creating an uncertain and complex clinical work environment. In the absence of clearly defined evaluation benchmarks and consistent feedback, participants reported that they had to resort to repeated self-assessment strategies, such as reflection, comparing their own practice with that of their colleagues, and mentally rehearsing possible alternative approaches, to determine whether they had met role standards.

“There are no clear standards or consistent feedback to tell me whether I am doing well or not. Most of the time, I can only rely on my own judgment to determine whether my approaches at work are acceptable.” *(Participant 7).*

“I often find myself repeatedly going over in my mind whether the judgments and interventions I made at the time were correct, and thinking about whether there might have been better ways to handle things, or whether I would still need to take similar approaches if I encountered a similar situation in the future...... I also frequently compare myself with others, because I am not sure whether what I have done is good enough.” *(Participant 13).*

#### 3.1.2 Uncertainty in work-related stress

As the demands of clinical IPC work continue to increase and the psychological burden intensifies, the stress experienced by participants in this study was not only escalating linearly but also had complex origins and was uncontrollable. IPCNs often cope with this uncertainty by investing more time, energy and attention. Participants also indicated that they simultaneously endured escalating psychological stress and rising work expectations as their work responsibilities continued to expand, consequently having to increase their personal investment to manage this situation. Participants also mentioned that this involuntary sustained investment enabled them to fulfill job demands during periods of escalating pressure.

“The workload has been increasing continuously, and I feel that the regular working hours are simply insufficient to handle all these tasks. As the pressure keeps mounting, I have no choice but to sacrifice my personal time and energy just to manage to complete the work tasks to a relatively satisfactory standard.” *(Participant 8).*

“Sometimes this is not a voluntary choice, but rather something I have to do. I need to be more focused and devote more personal time in order to meet the expectations of my supervisors and colleagues.” *(Participant 14).*

#### 3.1.3 Uncertainty in risk exposure

The ambiguous boundaries of IPCNs’ competencies and responsibilities at the study site led them to make judgments and decisions that put them at risk of errors in practice, due to a lack of clarity regarding their positional authority and responsibilities. In this study, participants said that they would still make judgments and implement interventions proactively, even when uncertain whether the issues in question fell within their remit. Participants also said that taking responsibility for making decisions about clinical IPC under such ambiguous competency boundaries meant they were alone in bearing the potential risks associated with their interventions, including the consequences of poor decisions or retrospective accountability.

“Sometimes I am not even sure whether this falls within my scope of responsibilities, but if I do not do it, no one else will, so I have no choice but to take the initiative.” *(Participant 6).*

“When making decisions, I am aware that there are potential risks behind those decisions, such as being held accountable by my supervisors for poor performance or causing adverse consequences for clinical patients and the hospital. However, I am very clear that I am the person who has to assume responsibility at that moment.” *(Participant 9).*

### 3.2 Challenges and costs in achieving competent practice

In the clinical context of IPC, IPCNs continuously faced difficulties in maintaining their professional competence while sustaining ongoing personal depletion. Furthermore, we identified and synthesized four subthemes closely related to this core theme, including “Unclear initial competence boundaries,” “Ambiguity in practical judgment,” “Role insecurity,” and “Sustained personal depletion.”

#### 3.2.1 Unclear initial competence boundaries

In terms of developing the professional competence of IPCNs, participants consistently demonstrated that, during the initial period of taking on this role, unclear role boundaries frequently led to uncertainty regarding their competence in the position. This uncertainty led participants to doubt their clinical decisions and professional judgments, resulting in a lack of confidence. Sometimes, this even led them to repeatedly evaluate whether they were ‘good enough’. As this ongoing cognitive struggle deepened, participants gradually developed emotional concerns such as anxiety, self-doubt, and stress.

“At the beginning, I was often unsure whether my decisions were correct. Since there were no clear documents specifying what fell within my scope of responsibilities, every judgment and decision I made left me feeling somewhat uncertain.” *(Participant 2).*

“I often found myself mentally replaying my previous decisions and judgments over and over, wondering whether they were correct and where I had fallen short. I kept thinking that an experienced senior would certainly have done better than me......I was constantly doubting myself—whether I was truly competent enough for this position.” *(Participant 7).*

#### 3.2.2 Ambiguity in practical judgment

In the context of unclear role boundaries and ill-defined responsibilities, IPCNs were often unable to accurately determine the appropriate timing for implementing interventions. However, rather than passively waiting for established rules, they gradually developed their judgment capabilities through clinical practice. Some participants said that, during clinical practice, they had tried to learn how to recognize the right moment to intervene proactively and make judgments, while also knowing when to step back and identify the optimal timing for intervention. Other participants described relying on personal experience, the reactions of clinical patients and staff, classic cases, and careful observation to make an initial, relatively objective judgment. Subsequently, they adopted the corresponding approach based on the severity of the event, such as providing proactive reminders, engaging in in-depth communication, reporting to superiors, or implementing emergency measures according to high-risk management protocols. As they gained more clinical experience, IPCNs’ practical judgment gradually improved.

“There were no clear regulations specifying when I should implement an intervention and when I should not. I could only feel my way forward......I learned more about judging the optimal timing to take action through the process of practice.” *(Participant 1).*

“I generally choose to step back appropriately when proactive intervention does not help solve the problem. I feel that this approach actually leads to better problem resolution......As practical experience accumulated, I gradually became able to grasp more opportune moments for taking action.” *(Participant 4).*

“I typically assess the severity of an event based on personal work experience, cases I have encountered or observed, patient reactions, and feedback from clinical colleagues......according to different severity levels, I adopt different management approaches......For instance, reminders and communication may suffice for lower-level issues, while serious cases require timely reporting and handling according to emergency protocols.” *(Participant 9).*

#### 3.2.3 Role insecurity

IPCNs’ sense of role security was sometimes destabilized when they felt their competence was insufficient to meet the role’s demands. In these moments, participants often questioned the correctness of their decisions and their overall fitness for the role. This insecurity was not constant but arose when participants’ sense of competence was challenged, and tended to diminish with accumulated experience or external support.

“There were situations I had never encountered before, which made me intensely concerned about whether I was handling them correctly......for example, whether my infection control decisions were appropriate and what the consequences might be if mistakes occurred......At times, I even questioned whether I had the capability and qualifications to undertake such a complex role.” *(Participant 8).*

“Although I sometimes meet totally new situations with no prior identical experience, my experience in dealing with similar cases, combined with support and guidance from senior colleagues, can alleviate my concerns.” *(Participant 11).*

#### 3.2.4 Sustained personal depletion

In the absence of institutional support, IPCNs kept operations running by sacrificing their time and effort. They even compromised some parts of their lives. This reduced the chances of mistakes and the anxiety regarding accountability, and helped them feel secure. Several respondents indicated that they frequently put work before their personal lives, spending significant personal time and effort on work sustainability and viability. Although their job roles could not be changed, this long-term personal investment helped them to obtain short-term security.

“I often work until midnight after official hours just to maintain the quality of my work and create a sense of security for myself.” *(Participant 2).*

“Although the nature of my job responsibilities remains largely unchanged, additional personal investment allows me to establish a sense of security.....By dedicating more effort to ensuring meticulous infection control practices, I reduce the likelihood of errors and potential criticism from supervisors.” *(Participant 11).*

### 3.3 Informal learning in the absence of institutional support

When formal training proved insufficient to meet the work demands of IPCNs, they compensated through informal learning in daily practice and implicit support from colleagues and the work environment. Corresponding subthemes include “Peer mentoring,” “Experiential learning,” “Compensatory competence development,” and “Developing a sense of competence through practice.”

#### 3.3.1 Peer mentoring

IPCNs in this study acquired professional competencies they can use in clinical infection control incidents by interacting with more experienced colleagues during clinical practice. The participants explained that, during their initial years in their respective positions, they had been highly dependent on peer mentoring whenever they were in new working environments and navigating complicated clinical situations. This type of mentorship, in which senior staff offer context-based advice, direction, and assessment to the novices, was internalized in their day-to-day work practices.

“Initially, when I was unfamiliar with the clinical work and scenarios of infection control nurses, I primarily acquired essential skills by learning from experienced colleagues.” *(Participant 6).*

“I feel that most of the practical guidance I received did not occur during formal training sessions, but rather when handling problems......experienced mentors would immediately point out and demonstrate how I should proceed.” *(Participant 12).*

#### 3.3.2 Experiential learning

This mentorship approach, in which experienced staff provide novices with context-specific advice, guidance and feedback, became an integral part of their daily work routines. Participants stated that they gradually learned and internalized infection control norms and practice standards by working alongside colleagues and sharing expectations in their daily practice. Additionally, participants indicated that they were more likely to comply with work standards and norms by observing how experienced colleagues handled IPC events in specific contexts than to adhere solely to written regulations. This approach was particularly valuable for guidance in situations lacking clear instructions.

“We do not rely on holding the standards and regulation documents directly to complete IPC tasks......we slowly learn and master them through daily collaboration with colleagues and the expectations placed on us at the clinical frontline.” *(Participant 2).*

“In the absence of clear and explicit standards and guidelines, observing how surrounding colleagues handle clinical IPC events provides me with the most direct and efficient guidance and assistance.” *(Participant 5).*

#### 3.3.3 Compensatory competence development

When IPCNs identified gaps in their professional competence, they compensated through three main strategies: self-directed learning, observation and imitation of others, and continuous trial and error in practice. Specifically, this involved proactively seeking relevant literature, official guidelines, or online training when encountering unfamiliar IPC events; observing and imitating the practices of more experienced colleagues in similar clinical settings; and iteratively refining their problem-solving approaches through trial and error, adjusting based on incident outcomes, others’ reactions, and environmental changes.

“When I realize that I lack the necessary knowledge to face new IPC situations, I usually need to engage in proactive learning to address the circumstances, such as consulting guidelines, searching for information online, and taking the initiative to participate in online training.” *(Participant 8).*

*In the face of similar situations, observing the measures and actions taken by experienced colleagues and applying them to my subsequent clinical practice has allowed me to learn a great deal.” (Participant 10).*

“In the beginning, I often made mistakes, but I would continuously correct them and adjust my strategies during practice to explore feasible coping methods as quickly as possible, thereby compensating for my lack of competence.” *(Participant 15).*

“Due to the absence of a clear evaluation system, I often need to rely on observing the reactions of clinical staff to judge whether my management methods are effective.” *(Participant 3).*

“I mainly rely on retrospectively reviewing the outcomes of IPC issues and observing the reactions of ward colleagues to determine whether the judgments and decisions I made at that time were appropriate.” *(Participant 16).*

#### 3.3.4 Developing a sense of competence through practice

Participants perceived that they gradually developed competence and confidence through repeated clinical practice. This subjective perception did not arise automatically, but was established through repeated exposure to similar situations. This was accompanied by increasing familiarity with managing HAIs incidents and a diminishing reliance on external validation, which manifested in more active and confident participation in real-world decision-making.

“At the beginning, I had very little confidence in myself, but after repeated exposure to and experience with similar clinical situations, I began to become familiar with what needed to be done and clear about my scope of responsibility, and slowly started to feel competent in this role.” *(Participant 5).*

“Unlike the beginning, I don’t need to repeatedly confirm with others whether what I am doing is right to boost my confidence. Because I am becoming increasingly confident in my professional capabilities......I am willing to actively participate in the handling and decision-making of infection control events.” *(Participant 11).*

### 3.4 Mutual shaping of professional competence and role identity

Competency serves as the cornerstone for the emergence, consolidation, and sustained stability of IPCNs’ role identity. This core theme encompasses three subthemes: “Competence experience-driven role identity transformation,” “Mutual reinforcement of competence accumulation and role identity,” and “Role identity destabilization upon competence setbacks.”

#### 3.4.1 Competence experience-driven role identity transformation

Practical experience is one of the most important factors in the formation of role identity among IPCNs. Participants noticed that certain pivotal moments in their practice, such as addressing complex infection control issues independently, making accurate decisions, and receiving recognition from clinical colleagues, constituted significant milestones in the development of their professional identity. Unlike the gradual process of formal accreditation, interviewees reported that their perception of becoming a genuine IPCN was predominantly based on concrete, practical experiences. Through these critical practice junctures, respondents truly began to identify themselves as incumbents of this professional role.

“There was an instance where I independently managed a complex clinical infection control issue with successful outcomes. It was at that moment that I felt completely competent in my role as an infection control nurse.” *(Participant 1).*

“When clinical infection control issues arose, and frontline colleagues proactively sought my assistance, trusting my professional judgment, I felt they genuinely perceived me as a specialized IPCN.” *(Participant 5).*

#### 3.4.2 Mutual reinforcement of competence accumulation and role identity

IPCNs’ sense of role identity was strengthened as they perceived themselves as increasingly competent and confident in executing their professional responsibilities, which led to a more stable identity that was less susceptible to fluctuation. Conversely, participants who developed a stronger role identity reported greater motivation to further improve their competencies to meet role expectations.

“As my tenure extended, with accumulated experience and deepening familiarity with job responsibilities, I grew increasingly confident and developed a more profound understanding of this role.” *(Participant 7).*

“Now I rarely experience the self-doubt I once had. My mindset has matured, and even when confronting challenging infection control issues, I can maintain a stable perception of my position within this role.” *(Participant 12).*

“When I truly identified myself as a professional infection control nurse, I became more motivated to enhance my competencies to better align with this role and meet collective expectations......I noticed accelerated skill acquisition and higher work efficiency during periods of strong professional identity affirmation.” *(Participant 15).*

#### 3.4.3 Role identity destabilization upon competence setbacks

When IPCNs believed they were not capable of performing their professional functions to the required extent, they could develop new uncertainties and role discontinuities. Respondents also indicated that they faced new questions about their role-specific skills and jurisdiction during complex, challenging, or highly demanding infection control tasks. Insecurity and self-doubt often accompanied this uncertainty. These situations showed that the role identity of the IPCNs depended on context, suggesting that the respondents saw their sense of role identity as directly connected to the perceived competence in the profession.

“Occasionally, when encountering highly unfamiliar or complex clinical scenarios, I begin to question whether I can adequately fulfil the scope of duties required by this role.” *(Participant 9).*

“Although I have experienced numerous clinical infection control situations, there are instances where I still feel unsettled and self-doubting, as I am uncertain whether my current capabilities are truly sufficient to manage them effectively.” *(Participant 13).*

## 4 Discussion

This qualitative research paper examines the formation and development of professional competence in the field of clinical practice of IPCNs in this study, without the influence of professional training and institutional promotion. The study found that IPCNs in this setting often struggled to rely on defined, consistent competency standards to meet their obligations. Instead, they relied on alternative approaches, including practical use of experience through trial and error and informal learning, to build their competence. The responses describing how they developed their professional competence as IPCNs indicated that their competence did not develop linearly through an integrated training program but spirally through work challenges, practice settings, and self-adaptation. This study enhances our understanding of IPCNs’ experiences in this practice setting in developing practice-based competencies in authentic clinical settings. It also provides a theoretical basis for creating contextually appropriate, position-specific training systems and sustained support mechanisms.

This study found that the routine working context for IPCNs in clinical practice is constituted by uncertainty structures across multiple dimensions, including role positioning, stress and risk. In the absence of clear role definitions and evaluation criteria, IPCNs often had to monitor themselves continuously and engaged in reflective practice to determine the appropriateness of their actions. However, a lack of external feedback and support can lead to over-reliance on self-monitoring, which can result in a cycle of self-doubt (31). This is consistent with this study’s findings. To mitigate this impact, organizations need to establish clearer evaluation criteria and provide the necessary support and feedback to help individuals have a more positive experience of self-monitoring and reflection processes (30, 32). This approach enables organizations to reduce self-doubt among IPCNs and foster healthier professional development environments, in line with Cheng et al. (31). Participants in this study also indicated that ambiguous delineation of responsibilities led to them involuntarily assuming responsibilities, a process which was often accompanied by significant psychological stress. Sørensen et al. (33) also suggest that responsibility ambiguity can make nursing staff feel uneasy and uncertain when dealing with complex situations. This affects not only individuals’ psychological state, but also their behavior. Furthermore, in contexts where competency boundaries and the scope of responsibilities remain unclear, IPCNs must make immediate judgments about IPC. This transferred practical risks, which should be shared by medical systems or teams, to the individual level, consequently exacerbating psychological stress. Moreal et al. (34) found that challenges in IPC activities cannot be attributed only to individual professional competence, but are also shaped by systemic and organizational issues, including unexpected rises in patient volume and urgency, a lack of human resources, and insufficient organizational support. Sist et al. (35) reached a similar conclusion, showing that problems in nursing practice are typically driven by the interplay of factors at the system, unit, nurse management, nurse, and patient levels. Taken together, these findings suggest that future capacity-building and support efforts should not focus solely on strengthening individual knowledge and skills, but should also address system-level support such as optimising staffing and resource allocation within departments, improving organizational processes and departmental support, strengthening leadership engagement and feedback mechanisms, offering more targeted development for younger or less experienced nurses, and continuing to refine how barriers to, and lapses in, IPC practice are identified and evaluated.

In this study, participants indicated that, in contexts characterized by a lack of institutionalized support, ill-defined role boundaries, and unclear responsibilities, they often had to develop their professional competence proactively through repeated clinical practice. This study reveals that the IPCNs did not benefit from clear and stable institutional support, but instead developed their professional competence gradually within highly uncertain clinical practice contexts. Participants therefore had to rely on methods such as their accumulated clinical experience and observations of responses from clinical patients and staff in practical contexts to determine the optimal timing and effectiveness of interventions and the most appropriate approaches. Multiple studies suggest that professional competence can be acquired through systematic training and education and assessed using structured, multidimensional competency frameworks (36–38). These frameworks have been shown to be relatively reliable and valid across diverse cultural contexts. However, this study’s findings reveal that, in uncertain healthcare contexts, IPCNs’ professional competence in this study did not stem from capabilities acquired *a priori* but formed gradually through clinical practice and remained in a state of dynamic evolution. Previous studies on healthcare professionals’ professional competence have predominantly been conducted in relatively stable healthcare contexts, relying on well-defined role responsibilities and evaluation criteria (39, 40). In these contexts, professional competence is regarded as the outcome of experiential accumulation or training. However, this study reveals that professional competence forms in a fundamentally distinct way. Specifically, in contexts with high uncertainty and a lack of institutionalized support, professional competence does not naturally emerge over time and with experience. Rather, it emerges when IPCNs make autonomous judgments and continuously adapt their practices in response to blurred role boundaries, constantly evolving responsibilities, and frequent potential risks. The uncertain circumstances described in this study should not be viewed simply as obstacles, but as key factors that influence the development of professional competence. The study by Holmes et al. (39) also showed that situational training can improve IPCNs’ core competencies in high-risk practices, strengthen their operational responsiveness and facilitate their transition towards autonomous practice. Therefore, given the current lack of institutionalization and unclear role boundaries, our study suggests that healthcare institutions should not focus only on reducing uncertainty or providing standardized procedures. Instead, they should incorporate such uncertain scenarios into routine training and support IPCNs’ professional development in similar practice settings through experiential reflection, summarisation, and case-based learning. They should also provide practice support to encourage sustainable adaptation. Additionally, this study indicates that, in contexts lacking systematic support and clear role boundaries, IPCNs must continuously invest their own time, energy, and emotional resources to fulfill their role responsibilities and achieve a sense of security. This self-consumption is not merely the result of individual choices that are partially involuntary, but rather a compelled coping strategy in response to structural constraints. It is also a critical approach to maintaining professional competence, which aligns with the findings of Maisonneuve et al. (41). Participants noted that IPCNs often maintained operational continuity in complex practice contexts at the expense of self-consumption. Therefore, it is important to consider how to reduce the hidden costs that individuals incur when developing professional competence by providing support and protective measures. While this approach of using individual resources to cope with uncertainties in practice ensures short-term continuity of role responsibilities and stability of professional competency, it also exposes systemic risks arising from deficits in career development support for IPCNs in this setting, as well as tendencies to individualize structural issues. Therefore, our study recommends that healthcare systems provide systemic support, such as transferring certain risks from individuals to organizations and institutions, rather than relying on individuals to maintain their role responsibilities through self-consumption. This would mitigate the implicit costs individuals currently bear while developing professional competency. Furthermore, such self-consumption could potentially threaten the psychological well-being and career sustainability of IPCNs. This is because the challenges faced by IPCNs in their roles extend beyond the technical to encompass the psychological and emotional aspects of self-consumption. Chan et al. (42) also showed that chronic psychological strain and emotional exhaustion can lead to professional burnout. This affects long-term career sustainability and patient safety. Tran et al. (43) reported on the mediating roles of emotional exhaustion and collaborative climate in healthcare workers’ work-life balance. Therefore, this study recommends that hospital management focus on providing psychosocial support to mitigate IPCNs’ self-consumption in complex contexts by enhancing collaborative teamwork and providing emotional support. This would improve both professional sustainability and patient safety.

Our study also revealed that, in the absence of formal, standardized training, participants proactively resorted to informal learning as a tacit support mechanism to meet complex clinical demands. Participants attributed their engagement in informal learning behaviors and pursuit of tacit support to the complexity and unpredictability of clinical infection control issues, ambiguous roles with unclear responsibilities, and an urgent need for targeted, real-time support. Furthermore, we found that these informal learning processes were not random, but constituted a tacit support network which encompassed the following: providing IPCNs with clinically-based, just-in-time guidance through peer mentoring; enabling the internalisation of job requirements and norms into personal consciousness via intra-departmental experience transmission; proactively addressing competency gaps through self-directed learning and modelling; and obtaining feedback coupled with behavioral adaptation based on practice outcomes and observed responses from others. In contrast to the perspectives of Al-Omary et al. (40) and Alasfour et al. (44), who regard informal learning as a means of complementing formal training for healthcare professionals, our findings suggest that, for the IPCNs in this study, informal learning served as the primary pathway through which participants perceived themselves as developing professional competence.

Self-directed learning (SDL) skills, as one form of informal learning, has been shown to play an important role in fostering professional growth and readiness for clinical roles and may cultivate autonomy and confidence (45). However, in this study, participants’ accounts reflect their perceived ability to manage IPC demands, rather than objectively measured competence assessed through independent, performance-based measures. In addition, Dentice et al. (46) similarly found weak positive correlations between perceived SDL skills and actual competencies, suggesting that self-perceived learning gains do not necessarily correspond to demonstrated performance. This can partly be explained by the fact that the translation of informal learning into practical competencies is itself shaped by systemic factors such as the clinical learning environment, mentorship, real-world experiences, and unit experiences (46, 47). Consequently, capacity-building initiatives for IPCNs should treat informal learning as an important source of confidence and adaptability, and invest in structured, performance-based assessment to determine whether such learning has been translated into actual competence. When opportunities for direct clinical practice are limited, targeted mentorship and compensatory training strategies may help support this translation from perceived to actual competence.

Additionally, this study revealed that, in practice-oriented healthcare contexts characterized by blurred role boundaries and institutional deficiencies, IPCNs’ professional competence is in a dynamic, context-dependent, reciprocal relationship with their role identity. Specifically, an individual’s sense of role identification is strengthened when their level of professional competence is reinforced. Conversely, their professional competence is undermined when their sense of role identification is shaken. Moreover, as professional contexts evolve, individuals’ perceptions of their own competence change continuously, while their sense of professional identity is simultaneously reinforced or undermined. Unlike Blum et al. (48), who posit role identity as the primary facilitator of professional competence, we argue that the relationship between IPCNs’ professional competence level and their role identity is interactive and repeatedly constructed across different practice contexts. Participants in this study indicated that, in familiar, predictable and relatively risk-controllable practice contexts, they could often establish a relatively stable sense of professional competence based on experience and mature judgment. This helped them to maintain a solid sense of role identity. However, even participants with extensive experience may experience doubts about their capabilities and role boundaries when confronted with unfamiliar, complex or high-risk clinical situations, leading to subsequent fluctuations in their professional role identity. This finding further suggests that the professional identity of IPCNs does not demonstrate stable or spiral progression with the accumulation of work experience. Instead, it undergoes continuous evolution through confirmation, challenge, and reconstruction in response to different practice contexts. Therefore, this study recommends that healthcare systems and administrators focus not only on enhancing IPCNs’ professional competencies but also on how they interpret their roles in different practice contexts. By providing reflective space, offering timely feedback at critical points in a healthcare professional’s practice, and recognizing their professional judgment, systems can alleviate the self-doubt and insecurity triggered when their competence is questioned. This enhances self-confidence and supports career development.

## 5 Limitations

This study had some limitations. Firstly, this study only recruited IPCNs from a single medical institution. Although theoretical saturation was achieved within this single-site sample, the relatively homogeneous institutional context constrains the diversity of practice environments and organizational conditions captured by this study, and this should be considered when considering the transferability of the findings. Secondly, in this study, professional competence in infection control practices was mainly developed through IPCNs, without input from other stakeholders, including hospital administrators, clinical departments, and healthcare service users. In turn, the discussion of the mechanism of multi-stakeholder collaboration in forming IPCNs’ professional competency is very poor. Thirdly, since interviews were conducted in Mandarin and only selected quotations were translated into English, some nuance may have been lost in translation, despite independent review of the translated material against the original transcripts. Despite the limitations mentioned above, future studies could involve more stakeholders and include different areas and healthcare facilities, which would help capture a broader range of perspectives and insights into how IPCNs develop and shape their professional competence across clinical settings.

## 6 Conclusion

Under the combined influence of subjective cognition and objective structural limitations, IPCNs in this study developed their professional competence in the increasingly complicated and unpredictable clinical scenario. Although they face role-boundary issues, limited institutional support, and unpredictable high risks, informal learning and peer mentoring, along with adaptive self-regulation, are crucial to developing professional competence, as shown by their clinical practice experience. These findings indicate an immediate need to implement competency-based training programmes, continuous provision and progressive certification to support IPC practice, alongside structured mechanisms for verifying competence, to better support IPC practice in similar tertiary-hospital settings.

## Data Availability

The original contributions presented in the study are included in the article/Supplementary material, further inquiries can be directed to the corresponding author.
