# Supplementary material for: Professional competence development in the context of clinical uncertainty: a qualitative exploration of infection prevention and control nurses at a tertiary hospital in China
Source: Front Public Health. 2026 Sep 7;14:1865842. doi: 10.3389/fpubh.2026.1865842 (PMC13590566; doi:10.3389/fpubh.2026.1865842)
Supplement: Supplementary file 1 [file Data_Sheet_1.pdf]

Consolidated criteria for reporting qualitative studies (COREQ): 32-item checklist

No	Item	Guide questions/description	Reported on page No
Domain 1: Research team and reflexivity			
1	Interviewer/facilitator	Which author/s conducted the interview or focus group?	2.3 Data collection
2	Credentials	What were the researcher's credentials?	2.3 Data collection
3	Occupation	What was their occupation at the time of the study?	2.3 Data collection
4	Gender	Was the researcher male or female?	2.3 Data collection
5	Experience and training	What experience or training did the researcher have?	2.3 Data collection
6	Relationship established	Was a relationship established prior to study commencement?	2.3 Data collection
7	Participant knowledge of the interviewer	What did the participants know about the researcher?	2.2 Participants
8	Interviewer characteristics	What characteristics were reported about the interviewer/ facilitator?	2.3 Data collection
Domain 2: study design			
9	Methodological orientation and Theory	What methodological orientation was stated to underpin the study?	2.1 Design
10	Sampling	How were participants selected?	2.2 Participants
11	Method of approach	How were participants approached?	2.2 Participants

Consolidated criteria for reporting qualitative studies (COREQ): 32-item checklist (Continued)

No	Item	Guide questions/description	Reported on page No
Domain 2: study design			
12	Sample size	How many participants were in the study?	2.3 Data collection
13	Non-participation	How many people refused to participate or dropped out?Reasons?	2.3 Data collection
14	Setting of data collection	Where was the data collected?	2.3 Data collection
15	Presence of non-participants	Was anyone else present besides the participants and researchers?	2.3 Data collection
16	Description of sample	What are the important characteristics of the sample?	Table 1
17	Interview guide	Were questions, prompts, guides provided by the authors? Was it pilot tested?	2.3 Data collection
18	Repeat interviews	Were repeat interviews carried out? If yes, how many?	2.3 Data collection
19	Audio/visual recording	Did the research use audio or visual recording to collect the data?	2.3 Data collection
20	Field notes	Were field notes made during and/or after the interview or focus group?	NO
21	Duration	What was the duration of the interviews or focus group?	2.3 Data collection
22	Data saturation	Was data saturation discussed?	2.3 Data collection
23	Transcripts returned	Were transcripts returned to participants for comment and/or correction?	2.5 Rigor

Consolidated criteria for reporting qualitative studies (COREQ): 32-item checklist (Continued)

No	Item	Guide questions/description	Reported on page No
Domain 3: analysis and findings			
24	Number of data coders	How many data coders coded the data?	2.4 Data analysis
25	Description of the coding tree	Did authors provide a description of the coding tree?	Not applicable
26	Derivation of themes	Were themes identified in advance or derived from the data?	2.4 Data analysis
27	Software	What software, if applicable, was used to manage the data?	2.4 Data analysis
28	Participant checking	Did participants provide feedback on the findings?	2.5 Rigor
29	Quotations presented	Were participant quotations presented to illustrate the themes / findings? Was each quotation identified?	3 Results
30	Data and findings consistent	Was there consistency between the data presented and the findings?	3 Results
31	Clarity of major themes	Were major themes clearly presented in the findings?	3 Results
32	Clarity of minor themes	Is there a description of diverse cases or discussion of minor themes?	4 Discussion

Note: P: page
